# The Vicious Circle of Working Hours, Sleep, and Recovery in Expert Work

**DOI:** 10.3390/ijerph15071361

**Published:** 2018-06-28

**Authors:** Annina Ropponen, Mikko Härmä, Barbara Bergbom, Jouko Nätti, Mikael Sallinen

**Affiliations:** 1 Finnish Institute of Occupational Health, P.O. Box 18, 00032 Helsinki, Finland; mikko.harma@ttl.fi (M.H.); barbara.bergbom@ttl.fi (B.B.); mikael.sallinen@ttl.fi (M.S.); 2 School of Social Sciences and Humanities, University of Tampere, 33014 Tampereen yliopisto, Finland; jouko.natti@uta.fi

**Keywords:** cross-sectional studies, smartphone, mobile applications, sleep, leisure activities, time factors

## Abstract

This study aimed to investigate working hours, sleep quality and alertness, and recovery and detachment in expert work using a mobile app. The study sample comprised members of The Finnish Business School Graduates and employees of an information technology (IT) company. The final study sample included 154 employees with at least four days of mobile app data. For statistical analyses of the survey and mobile app data (cross-sectional setting), we used logistic regression, and for the day-to-day data, we used multi-level logistic regression to calculate the odds ratios (OR), and a general equation estimates model for regression coefficients with 95% confidence intervals (CI). The results showed that moderate to fair sleep quality or alertness at awakening were associated with longer working hours the following day (OR 1.07–1.14, 95% CI 1.01–1.22). Recovery and detachment during the preceding day were associated with longer working hours. These associations were the same in the opposite direction. To conclude, the day-to-day ratings of sleep quality and alertness at awakening, and recovery and detachment from work during leisure time were associated with increased working hours the following day. In addition, longer working hours the preceding day were associated with worse ratings of sleep quality, alertness, recovery, and detachment.

## 1. Introduction

Expert work today consists of knowledge work related to the development of the industrialized world. Digitalization has made expert work more common [1]. Expert work can be defined as requiring extensive formal education and continuous on-the-job learning, but also includes transferable skills. In expert work, the nature of work includes abstract knowledge and symbols (e.g., design and planning of production processes), while the organization of work ranges from professional bureaucracies to self-managing teams, and job and task circulation, but knowledge is a primary production factor [2]. The flexibility of expert work, through the use of mobile technology, has made it possible to work not only in offices, but also while travelling, at customers’ premises, in public places, or remotely [3,4]. Being able to influence working hours, i.e., individual flexibility, is generally linked to better health, wellbeing, and work/life balance [5,6,7]. Flexibility also means more autonomy for employees in terms of choosing when and where to work and which tasks to take on [8]. Hence, flexibility may lead to blurred boundaries between work and leisure time, and consequently to prolonged working hours [4,9].

Work organization (i.e., working hours, timing of work, and stress related factors) plays an important role in the associations between expert work, health, and wellbeing [5,10]. For example, working hours merit special attention since working hours is one of the few aspects of working conditions that has been regulated by national governments and labor unions, but also varies both within, and between, jobs and impacts wellbeing [11]. Therefore, individual characteristics of employees, work, and the organization of work potentially influence the increase of job demands. Hence, attention should be paid to working hours and well-being to clarify the factors, such as work hour control and flexibility, non-work demands, age, gender, job complexity, job demands, and norms that may potentially affect the effects of work on health and wellbeing [10,11]. Many of the findings regarding the effects of expert work on health and wellbeing have been mixed, i.e., flexibility may promote as well as compromise health and wellbeing [5,6,12]. These mixed findings are partly due to the fact that schedules for work tasks set by the employee themselves may prove to be unrealistic, leading to an increased workload and eventually to negative consequences for health, wellbeing, and the work/life balance [6]. Another explanation for the mixed findings on health and wellbeing in expert work may be related to remote work (i.e., working elsewhere than the office, nevertheless in close contact with one’s supervisor and colleagues), which may also lead to increased time pressure and stress due to a lack of support and contact, or unclear expectations of results [4].

The boundaries between work and leisure time are important for recovery and sleep. Placing boundaries on work enables detachment from work during leisure time, which is consequently important for recovery, especially when an employee has a strong commitment to work. On the other hand, employees with strong commitment to work perceive more energy and less loading after the working week [13,14]. One assumption is that high commitment to work may lead to longer working hours. An English study found that long working hours were associated with worse sleep quality among ministers [15], but a large-scale international study [16] showed that shortening working hours from 8 to 6 h a day had positive effects on sleep quality and alertness [17]. Indications exist that show a high workload may lead to difficulties in falling asleep [18], whereas sleep promotes recovery in many ways [19]. A recent meta-analysis showed that extending working hours negatively affected sleep length and quality, whereas work engagement improved sleep length and quality [20]. However, the association between work engagement and working hours has rarely been investigated [21]. A wider underlying assumption related to wellbeing and health is that recovery and detachment from work may prevent burnout [22], although the association with working hours in expert work remains to be shown [11].

Until now, most studies of working hours, recovery, and sleep in expert work have relied on retrospective and short-period self-reported surveys [4,23], or even on a few days utilizing diary methods [24]. Studies that have utilized daily-based information, focusing mainly on modelling work and physical activity at leisure-time [25,26] or flexible working hours [27], are relatively scarce. Hence further studies on day-to-day information regarding working hours, recovery, and sleep are warranted to shed further light on their role in wellbeing in expert work.

The aim of this study was to investigate the association between the use of daily time in expert work and sleep and recovery, utilizing a mobile app and survey data. We specifically aimed to study the day-to-day associations of sleep quality, alertness, recovery, and detachment with working hours.

## 2. Materials and Methods

The study sample included employed members of the Finnish Association of Business School Graduates and employees of an international information technology (IT) company. Participation in the study was voluntary and required the installation of a mobile application (mobile app) onto one’s smartphone. Altogether 1544 employees installed the mobile app and gave their approval for participation to the study. A total of 307 employees responded to the survey. Time use was recorded by 651 employees, and of these, 218 (14% of those with a mobile app and 33% of those with time use data) responded to the survey. The final study sample included 154 employees with at least four days’ worth of time use data and survey data. Due to a relatively large loss of data, we compared the survey respondents to those with only the mobile app data. We found no differences in terms of age, sex, or the survey data of time use (for example working hours/week) between those with only survey data, those with survey and mobile app data, or those with only mobile app data.

The survey consisted of comprehensive questions on work-related and sociodemographic factors. We elicited responses regarding age, sex, workplace size (categorized variable with response alternatives 1, 2–9, 10–29, 30–99, 100–249, 250–499, 500–999, or ≥1000), being a supervisor, and sector (with response alternatives: private sector, public sector, civil service, non-governmental organizations, or university).

We evaluated survey items regarding work-related mobile phone use outside regular working hours using a five-item measure with a five-point scale (from strongly disagree to strongly agree). The scale range was 5–25, and high values indicated a high degree of work-related mobile phone use outside regular work hours, as has been described in detail earlier [28]. We used the survey of the Standard Shiftwork Index [29] to evaluate sleep and alertness, which includes the following question: ‘Do your working hours affect your sleep and alertness?’ with the following response alternatives: negatively (often or somewhat), no, and positively (often or somewhat). Wellbeing was elicited by asking: ‘Do your working hours affect your wellbeing at work?’ with the same response alternatives as above for sleep and alertness.

Time use data were collected using a mobile app 24/7 with five preset dimensions: work, work without pay, leisure-time activities, physical activity, and sleep. During recruitment, the participants, were instructed to record their time use for 14 days in as much detail as possible, using the preset dimensions. We estimated also the midsleep time [30] based on the bedtime recorded by the mobile app. Hence, we lacked control for time to fall asleep which means that sleep onset and offset merely reflect the time in bed. Still, midsleep time has been considered useful for assessing chronotype [30] and an estimate for melatonin onset [31]. Furthermore, the mobile app collected data on sleep quality and alertness in the morning, i.e., at awakening, and recovery and detachment from work in the evenings, i.e., before going to bed.

Questions on sleep quality and alertness were based on the Pittsburgh Sleep Diary [32], and the recovery and detachment questions on the Recovery Experience Questionnaire, with response alternatives being fully agree, somewhat agree, do not know, somewhat disagree, and fully disagree [33]. These were surveyed passively, which means that the mobile app did not remind the participants of these, but the questions were recorded only if the participant logged in to the mobile app in the morning (time period between 05:00 and 10:00) and in the evening (time period between 18:00 and 22:00). For statistical analyses, both sleep quality and alertness, and recovery and detachment, were collapsed into three categories (good, moderate, fair; agree, do not know, disagree).

A third aspect of the mobile app was the possibility of collecting the perceived importance and perceived feelings related to the tasks recorded into the main dimensions. The three-point scale for importance and feelings was ‘good’, ‘neutral’ and ‘bad’.

### Statistical Analyses

Standard descriptive statistics including means with standard deviations and percentages were calculated for survey and mobile app data. First, utilizing cross-sectional design, we tested the associations between time use (mobile app data) and the indicators of wellbeing, sleep, and alertness (survey data), using logistic regression analyses for odds ratios (OR) and 95% confidence intervals (CI), controlling for age and sex. We tested the effect of workplace size, being a supervisor, sector, perceived importance or feelings, and work-related mobile phone use during off-hours on the associations. However, as their (separate) effect was minimal, i.e., all the associations remained in terms of magnitude and direction, we do not report these results here (data not shown). We also tested the effect of sleep length. The second set of analyses were based on day-to-day data gathered by the mobile app only. For statistical analyses, the day-to-day characteristics collected by the mobile app were treated in two ways. First, we evaluated the day-to-day characteristics during the recorded days for each individual. The sleep length and working hour characteristics, ratings of sleep quality and alertness, recovery and detachment, and perceived importance and feelings were averaged over all the days recorded by the mobile app. We also created a variable for indicating if an employee had worked in the evenings (between 18:00–23:00), as dichotomous yes/no, and another variable to indicate days on which the employee had worked for several periods. A break of at least two hours (leisure-time activities or physical activity) between two periods of work, or work without pay was the limit to being interpreted as several working periods. The number of recorded days with at least one recorded time period on the mobile app varied between 1 and 70. In order to maximize the number of employees in the dataset, but to capture variation between days, we set a criterion of four days as the minimum for being included in the further within-subject analyses. Due to within correlation and repeated measures, the day-to-day data were analyzed using multi-level logistic regression models, which is a regression method applicable to clustered data. This estimated odds ratios (OR) with a 95% CI. For these analyses, we collapsed the original five-point response scales of sleep quality and alertness, and recovery and detachment into a dichotomy by combining fully agree and somewhat agree into ‘agree’, and all the other three alternatives into ‘disagree’. In the statistical models, the groups with agree were used as a comparison group, i.e., we estimated the effect of mean working hours/day on perceived disagreement in sleep quality and alertness, and recovery and detachment. For a continuous day-to-day variable (working hours/day), we applied the general equation estimates (GEE) model to account for variation in correlation structures and inter-dependent observations within individuals and estimated regression coefficients with 95% CI. The effect of midsleep time was tested in the models.

This study was granted ethical permission by the ethical committee of the Finnish Institute of Occupational Health.

## 3. Results

Table 1 shows the characteristics of the study sample, and includes time use, sleep, alertness, and wellbeing. The age range was 19–62 years, slightly over half were men, and almost half of the sample reported that working hours often or somewhat affected their sleep, alertness, or wellbeing.

The day-to-day ratings of sleep quality and alertness, and recovery and detachment indicated that on average, 65–75% perceived their sleep quality and alertness as good at awakening (Table 2). Regarding recovery and detachment, on average 35–43% reported forgetting their work or not thinking about their work during leisure time, whereas 57–63% reported being able to distance themselves or take a break from work during their leisure time (on average).

The cross-sectional analyses yielded statistically significant associations between mean positive feelings at work and sleep quality and alertness (OR 0.37, 95% CI 0.18, 0.80), and between mean positive importance of work and wellbeing (OR 0.30, 95% CI 0.10, 0.91, Table 3). However, when sleep length was accounted for in the models, the association between working hours and wellbeing (OR 1.36, 95% CI 0.92, 2.02), and the association between working hours and sleep quality and alertness (OR 1.45, 95% CI 0.94, 2.21), became insignificant. All the other associations retained the magnitude and direction of the models in which only age and sex was accounted for (Table 3).

The associations between the day-to-day ratings of sleep quality and alertness and working hours/day showed that moderate to fair sleep quality or alertness at awakening were associated with a higher likelihood of longer working hours the following day (Table 4). In addition, a lack (do not know/disagree) of recovery and detachment from work during leisure time the preceding evening was associated with an increase in working hours the following day. These associations shown in Table 4 retained their direction and magnitude even when the working hours of the preceding day or midsleep time were controlled.

**Figure 1:**
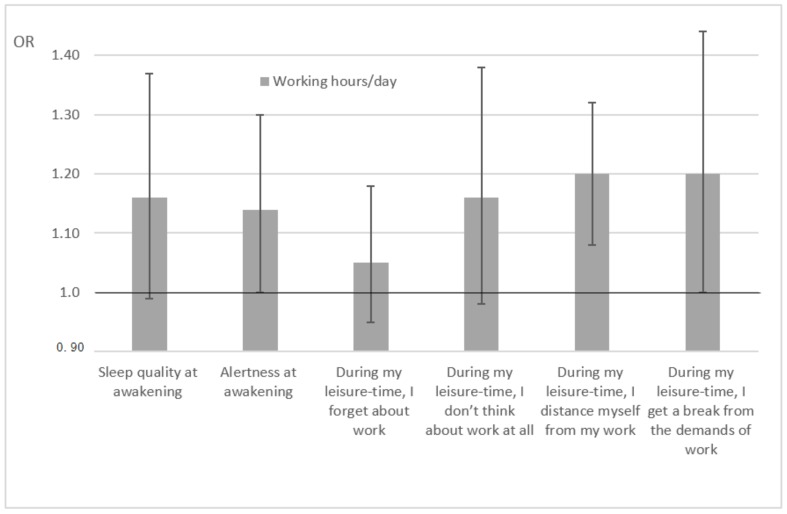
Odds ratios (OR) for association between working hours/day and ratings of sleep quality and alertness, and recovery and detachment (midsleep time accounted in the analysis). Error bars indicate 95% CIs.

The opposite direction in the day-to-day ratings of sleep quality and alertness, and recovery and detachment showed that longer working hours the preceding day were associated with a greater likelihood of worse sleep quality and alertness ratings at awakening (Figure 1). The trend was also similar for recovery and detachment, although not all the associations yielded statistical significance (95% CIs below 1.00 as indicated by the horizontal line in Figure 1). In the Figure 1, midsleep time has been accounted for and the crude associations were in the same direction and at the same magnitude although with a slightly tighter 95% CI (data not shown).

## 4. Discussion

This study of 154 employees in knowledge-intensive expert work in various sectors aimed to investigate daily time use in association with sleep and recovery utilizing a mobile app and survey data. This evaluation of the day-to-day associations between sleep quality and alertness, and recovery and detachment, and working hours might be among the first. The majority of the participants reported good sleep quality at awakening, whereas alertness was good among 65% of participants, although earlier findings have indicated that sleep quality might be compromised in expert work [15,20]. Thinking about work-related matters during leisure-time (48–57%) and not being able to detach from work during leisure time (24–33%) were relatively common, and were in line with earlier studies of experts [13]. Our cross-sectional analyses showed that positive feelings at work were associated with a lesser likelihood of worse sleep quality and alertness, and positive importance of work was associated with wellbeing at work. Although these were only cross-sectional associations, they point in the same direction as earlier studies of commitment to work being supportive of energy and less loading at work [13,14]. The specific interest of this study, namely day-to-day associations, indicated a vicious circle between sleep quality and alertness, detachment and recovery, and working hours. This study might be among the first to show that these associations exist in both directions, i.e., from a day of work to the next morning, but also from the preceding evening and morning to the next day. An earlier study of unreasonable and unnecessary tasks [26], a day-level study of recovery and work performance [34], and a review of recovery from job stress [35] have indicated the same trend. Furthermore, controlling the potential effect of chronotype [30] through the use of midsleep time in the analyses of the day-to-day associations between working hours and sleep quality, alertness, detachment, and recovery, confirmed the findings.

Based on the existing literature, we expected to see associations between time use and wellbeing or sleep quality and alertness [4,5,6,9,12]. However, perhaps due to the limited sample size or to cross-sectional analyses, we were not able to yield any statistically significant results for time use and wellbeing. Instead only feeling positive at work and positive importance of work were associated with sleep quality and alertness, and wellbeing, respectively. Although this finding merits caution, it may be in line with studies of work engagement improving sleep length and quality [20].

Day-to-day ratings and diary studies of time use [24], and recovery and detachment [36], have been relatively rare. Although day-to-day sleep studies are numerous [13,37], they usually also utilize other tracking methods, including wearable technology [38] or ambulatory techniques [39]. More specifically, associations with working hours on a day-to-day basis have been scarcely investigated. Hence, although the sample size and the limitation regarding the number of consecutive days (four days in minimum) must be borne in mind, the results of our study add to the knowledge on working hours, sleep quality and alertness, and detachment and recovery. More specifically, the potential to control midsleep time as a proxy for chronotype ascertained our findings pointing into direction that the association with working hours and sleep and alertness, but also with detachment and recovery is important for all. Not only further studies, but also workplaces and their supervisors, teams, and employees, as well as occupational health services would benefit from recognizing how working hours affect sleep quality and alertness, or recovery and detachment, as well as how they work in the opposite direction.

Although this study was limited to 154 employees, this study of working hours, sleep, and recovery in expert work might be of interest for those linked with work organizations, such as human resource departments, supervisors, or in occupational health care. Working hours are the aspect of working conditions that has been regulated by national governments and labor unions, and also often assessed at workplaces [11]. Our findings of the vicious circle between working hours, sleep, and recovery could be used for informing employees and supervisors to discuss possibilities regarding setting boundaries and to take care of leisure-time. Furthermore, since working hours varies both within, and between, jobs and consequently impacts wellbeing [11], perhaps both workplaces and occupational health care could consider early identification of those being at risk for excess amounts of work within certain time periods. Third, due to individual characteristics of employees, work, and the organization of work, instructions for possibilities to control working hours, or to add flexibility, but also how to react for non-work demands, in various age groups, and among both sexes would be needed in the workplaces, but also potentially at societal level too [10,11].

Despite the strengths of the study, i.e., utilizing the day-to-day mobile app data for time use, it also has some limitations. We aimed to gather data for 14 consecutive days, but only 154 participants had data from at least four days (range 4–70 days). Two weeks would have been optimal for obtaining data for two weekends, but we decided to use the four-day limit in order to maximize the number of participants. This may have resulted in some selection-bias that we cannot rule out. We assume that the lack of time use data was because this mobile app was free and not directly linked to any workplaces or their time control of working hours. Hence, we expected that those with survey data only would be different from those with mobile app data, but this was not the case. We cannot rule out that some selection bias may have affected our results, as the mobile app required a new smart phone and some familiarization with the use of mobile apps. Similar assumptions have been considered in earlier studies using various data collection methods [40]. Another limitation is related to the fact that we invited some 29,000 employees to participate in this study, but allowed only some 1500 of them to download the mobile app. This is a very low number, but the use of both cross-sectional and day-to-day within-subject analyses of time use, sleep quality and alertness, and recovery and detachment enabled sufficient statistical power. However, due to the low participation rate, and even the low number of subjects, we agree that further studies with even larger sample sizes would be merited to confirm these results.

Although this study only covered Finland, we were able to investigate employees in knowledge-intensive work in various sectors. Hence, we believe that these findings could be generalized to other industrialized countries in which expert work is emerging and that provide various possibilities for flexibility in terms of where and when to work [1,3,23].

## 5. Conclusions

Day-to-day ratings of sleep quality and alertness at awakening, and recovery and detachment from work during leisure time were associated with increased working hours the following day. In addition, vice versa, longer working hours the preceding day were associated with worse ratings of sleep quality and alertness, as well as worse recovery and detachment. This suggests a vicious circle, although further studies should confirm this result with larger sample sizes and longer follow-ups.

## Figures and Tables

**Table 1:** Descriptive statistics for background, time use, sleep, alertness, and wellbeing of 154 individuals.

Time Use Data Collected by Mobile App and Survey Data	Mean	SD
Age	41.7	0.5
Work-related mobile phone use during off-hours (range: 5–25)	18.8	6.9
Mean working hours/day	7.4	2.9
Mean working hours without pay/day	2.2	2.5
Mean sleeping hours/day	8.3	2.7
Mean midsleep time (o’clock)	3:07	1:20
Mean feeling at work/day (range: bad, neutral, good)	2.4	0.6
Mean importance of work/day (range: bad, neutral, good)	1.6	1.1
	**%**	
Sex (women)	42%	
Works evening hours (18:00–23:00, yes)	12%	
Working day includes several periods of work	30%	
Working hours affect sleep and alertness (often/somewhat)	59%	
Working hours affect wellbeing (often/somewhat)	44%	

**Table 2:** Percentages of day-to-day ratings of mean sleep quality, alertness, recovery, and detachment from work.

Time Use Data Collected by Mobile App/Day-to-Day Data	%
Sleep quality at awakening	
Good	75
Moderate	15
Fair	10
Alertness at awakening	
Good	65
Moderate	25
Fair	11
During my leisure time, I forget about work	
Agree	43
Do not know	9
Disagree	48
During my leisure time, I don’t think about work at all	
Agree	35
Do not know	8
Disagree	57
During my leisure time, I distance myself from my work	
Agree	57
Do not know	11
Disagree	33
During my leisure time, I get a break from the demands of work	
Agree	66
Do not know	10
Disagree	24

**Table 3:** Odds ratios (OR) with 95% confidence intervals (CI) for associations between time use and wellbeing and sleep in expert work.

Cross-Sectional Analyses between Time Use Data and Survey Data	Poor Wellbeing ^§^		Poor Sleep and Alertness ^§^	
	OR *	95% CI	OR *	95% CI
Mean working hours/day	1.06	0.90, 1.25	1.08	0.92, 1.27
Mean working hours without pay/day	0.90	0.69, 1.17	0.88	0.67, 1.15
Mean sleeping hours/day	1.05	0.71, 1.54	0.82	0.55, 1.22
Work during evening hours (18:00–23:00)	1.45	0.81, 2.59	1.71	0.93, 3.16
Working day includes several periods of work	1.04	0.50, 2.15	1.34	0.63, 2.85
Mean positive feeling during work/day	0.48	0.22, 1.03	**0.37**	**0.18, 0.80**
Mean positive importance of work/day	**0.30**	**0.10, 0.91**	0.60	0.23, 1.56

^§^ in comparison to those who had neutral, good, or very good wellbeing and/or sleep and alertness. * age and sex-adjusted model, statistically significant results in bold face.

**Table 4:** Regression coefficients with 95% confidence intervals (CI) for associations between day-to-day ratings of sleep quality and alertness, recovery and detachment, and working hours in expert work.

	Working Hours/Day			
	Crude Model		Adjusted Model *	
	Regression Coefficient	95% CI	Regression Coefficient	95% CI
Sleep quality at awakening (reference good)				
Moderate	1.02	0.96, 1.08	**1.09**	**1.01, 1.18**
Fair	**1.14**	**1.07, 1.22**	1.09	0.98, 1.20
Alertness at awakening (reference good)				
Moderate	**1.07**	**1.01, 1.12**	**1.08**	**1.01, 1.16**
Fair	**1.09**	**1.01, 1.17**	1.04	0.93, 1.16
During my leisure time, I forget about work (reference agree)				
Do not know	1.05	0.97, 1.14	1.04	0.94, 1.16
Disagree	1.04	0.99, 1.09	1.03	0.97, 1.09
During my leisure time, I don’t think about work at all (reference agree)				
Do not know	**1.09**	**1.00, 1.19**	**1.14**	**1.01, 1.29**
Disagree	**1.06**	**1.00, 1.11**	**1.07**	**1.00, 1.13**
During my leisure time, I distance myself from my work (reference agree)				
Do not know	**1.09**	**1.01, 1.17**	1.04	0.92, 1.17
Disagree	**1.06**	**1.01, 1.11**	**1.09**	**1.03, 1.16**
During my leisure time, I get a break from the demands of work (reference agree)				
Do not know	**1.12**	**1.04, 1.21**	1.08	0.97, 1.21
Disagree	**1.09**	**1.03, 1.15**	**1.07**	**1.00, 1.15**

* Adjusted for midsleep time. Bold figures indicate statistically significant results.
